# Covalent functionalisation controlled by molecular design for the aptameric recognition of serotonin in graphene-based field-effect transistors†

**DOI:** 10.1039/d3nr04153k

**Published:** 2023-09-19

**Authors:** Cecilia Wetzl, Sergi Brosel-Oliu, Marco Carini, Desiré Di Silvio, Xavi Illa, Rosa Villa, Anton Guimera, Elisabet Prats-Alfonso, Maurizio Prato, Alejandro Criado

**Affiliations:** a Center for Cooperative Research in Biomaterials (CIC biomaGUNE), Basque Research and Technology Alliance (BRTA) Paseo de Miramón 194 20014 Donostia-San Sebastián Spain mprato@cicbiomagune.es; b University of the Basque Country, UPV-EHU 20018 San Sebastián Spain; c Instituto de Microelectrónica de Barcelona, IMB-CNM (CSIC), Campus UAB Bellaterra Spain elisabet.prats@csic.es; d Centro de Investigación Biomédica en Red de Bioingeniería, Biomateriales y Nanomedicina, Instituto de Salud Carlos III Spain; e Ikerbasque, Basque Foundation for Science Bilbao Spain; f Dipartimento di Scienze Chimiche e Farmaceutiche, Università degli Studi di Trieste Trieste Italy; g Universidade da Coruña, CICA – Centro Interdisciplinar de Química e Bioloxía Rúa as Carballeiras 15071 A Coruña Spain a.criado@udc.es

## Abstract

In the last decade, solution-gated graphene field effect transistors (GFETs) showed their versatility in the development of a miniaturized multiplexed platform for electrophysiological recordings and sensing. Due to their working mechanism, the surface functionalisation and immobilisation of receptors are pivotal to ensure the proper functioning of devices. Herein, we present a controlled covalent functionalisation strategy based on molecular design and electrochemical triggering, which provide a monolayer-like functionalisation of micro-GFET arrays retaining the electronic properties of graphenes. The functionalisation layer as a receptor was then employed as the linker for serotonin aptamer conjugation. The micro-GFET arrays display sensitivity toward the target analyte in the micromolar range in a physiological buffer (PBS 10 mM). The sensor allows the in-flow real-time monitoring of serotonin transient concentrations with fast and reversible responses.

## Abstract 2

This work introduces a controlled covalent functionalization using molecular design and electrochemical triggering, enabling monolayer-like functionalization of GFET while preserving graphene's electronic properties for monitoring of serotonin.

## Graphical abstract

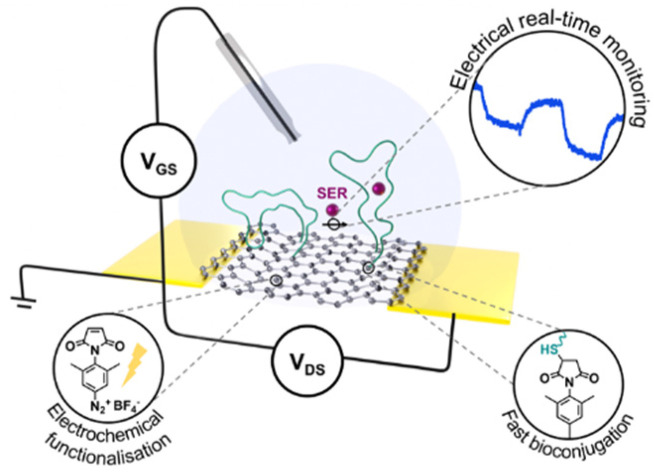

## 1. Introduction

In the last decade, solution-gated field effect transistors (FETs) have gained increasing interest as innovative platforms to develop all kinds of sensors, ranging from environmental analysis to biomarker monitoring.^1^ The reason for their success resides in features, such as intrinsic amplification capability, low sensitivity to environmental interferences and easy miniaturization, which make FETs suitable for developing cutting-edge electronics. The application of graphenes in electronic devices has been widely investigated due to their high carrier mobility.^2–9^ Compared to the commonly used metal oxide semiconductor field effect transistors (MOSFETs), graphenes add some crucial properties, such as transparency, biocompatibility, flexibility, and compatibility to most of the fabrication processes, to the final device.^10^ Additionally, compared to back gated devices, they possess higher sensitivity to voltage variations due to their higher gate capacitance and transconductance.^11^ Recently, graphene FETs (GFETs) have been employed to build flexible microtransistor arrays to perform electrophysiological signal recording *in vivo*.^12–16^ Owing to their operational method and structure, these devices can be applied in high-resolution brain mapping and disease monitoring. Additionally, GFETs proved to be outstanding platforms to develop specific biosensors selective to a wide variety of analytes (*e.g.*, protein or small molecules) operating also *in vivo*.^9,17,18^ The specificity toward different analytes can be pursued, including receptors *via* the functionalisation of the device. This last point is, up to date, probably the main issue that prevents the obtention of stable, reproducible GFET sensors. The functionalisation strategy employed must fulfil requirements, such as (i) long-lasting stability, (ii) fast bioconjugation with the receptor, (iii) preserved electronic properties, and (iv) control over the linker length. Even if graphene chemistry advance offered many valid strategies to this purpose, not all of them can be easily adapted to transistor modification. The most used modifications are indeed based on non-covalent chemistry since these strategies ensure the preservation of the electronic structure of the graphene and its high carrier mobility.^19,20^ By contrast, non-covalent strategies are poorly controllable with no possibility of reactivity tuning. However, the weak forces involved (1–5 kcal mol^−1^) in non-covalent interactions^21,22^ prejudice the long-term stability of functionalisation, being restrictive for *in vivo* platform development.^20^ Even though covalent chemistry is the most stable (∼85 kcal mol^−1^) and efficient way to functionalize graphenes, it gained the reputation of being completely useless to produce functional GFETs due to the high efficiency and poor control of the reaction. Currently, only a few works employed the most classical covalent functionalisation based on aryl diazonium salt to develop functional GFET sensors. During this kind of functionalisation, a highly reactive aryl radical is generated, which then reacts with graphenes or with other aryl moieties already attached on graphenes, ending with the so-called dendritic growth of the functionalisation layer.^23^ Both high reactivity and uncontrolled functionalisation layer growth must be avoided when developing a GFET sensor. In fact, while the disruption of the lattice obviously creates obstacles in the proper running of the device, the distance between the graphene and the receptor is also a crucial factor for the sensor functioning due to the Debye screening effect. As many researchers pointed out, the Debye screening effect is possibly one of the greatest limitations to the application of this device architecture to sensing, thus many efforts have been made trying to circumvent this problem.^24^ Up to date, the most successful strategies are focused on (i) shortening the receptor design, (ii) employing deformed graphenes, (iii) performing measurements in a low-ionic strength solution, or (iv) employing porous polymeric matrices permeable to the analyte.^25–27^

It is worth noting that Szunerits *et al.* proposed to control covalent grafting and obtain functional GFET-based cardiac troponin sensors using a protected aryl diazonium salt to induce monolayer-like functionalisation.^25,28,29^ The use of bulky groups that hinder the disruption of graphene lattices was extensively studied by the work of De Feyter's group, where the fine-tuning of the graphene functionalisation was obtained by grafting aryl diazonium salts bearing *tert*-butyl groups by electrochemistry.^23^

**Fig. 1:**
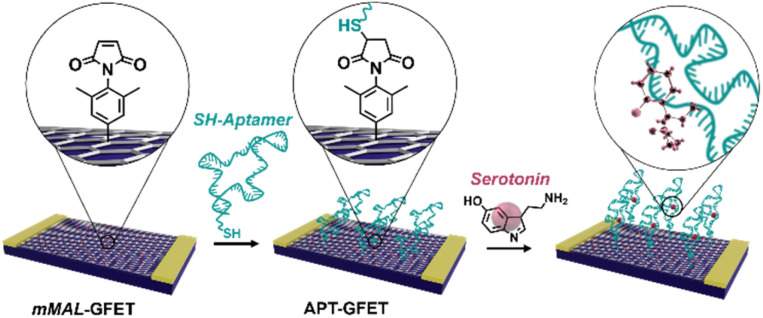
Schematic of the aptamer conjugation on functionalized GFETs (mMAL-GFETs) and subsequent serotonin recognition.

In this work, we developed a new strategy based on molecular design and electrochemical triggering to functionalize micro-GFET arrays in a controlled and stable manner, obtaining a monolayer-like functionalisation. Our synthesized diazonium salts, bearing a maleimide moiety, were then employed as linkers to anchor our receptor, a serotonin stem-loop aptamer (Fig. 1). The peculiarity of this oligonucleotide chain family is the capability of selectively binding serotonin, with a dissociation constant of 30 nM,^30^ and then change their conformation upon recognition. The so-produced conformation allows for the detection of small molecules by charge-sensitive devices such as GFETs. Indeed, the transduction methodology of these devices is based on the so-called field effect, thus the capability of the channel material (*i.e.*, graphene) of changing its electrical conductivity upon the application of an external electric field. Accordingly, while the detection of charged macromolecules as proteins is relatively easy, the recognition of small molecules is still an open challenge. This receptor has already been tested in different sensor architectures such as metal oxide FETs^31^ and glass nanopipettes^32,33^ to enable the fast and reversible real-time monitoring of the neurotransmitter. Additionally, similar oligonucleotides have been recently employed to develop a GFET-based sensor for dopamine and serotonin.^20,34^ This proof of concept validated the possibility of employing covalent functionalisation and aptameric recognition for the co-detection of dopamine and serotonin under physiological conditions. To obtain a functional device for the real-time neurotransmitter monitoring, still some further implementations are required. Among the others, the use of a multichannel array working in flow conditions and capable of recording in a reversible manner has not yet been reported.

In this work, we aimed to go a step closer to the obtention of a new platform for biomarker real-time monitoring with enhanced performances combining our innovative and controlled covalent functionalisation strategy, the micro-GFET array technology and the aptameric recognition system.

## 2. Results and discussion

### Molecular design functionalisation features

Aryl diazonium salts are among the most common reagents to functionalize graphenes in an efficient and stable way.^35,36^ The reaction proceeds *via* the decomposition of the diazonium moiety induced by the interaction with the electron-rich graphene lattice. This interaction generates a highly reactive aryl radical that subsequently reacts with graphenes or with other aryl moieties already attached on graphenes, ending with the so-called dendritic growth of the functionalisation layer.^23^ Both the high reactivity and the uncontrolled functionalisation layer growth are detrimental to the GFET sensor functioning. In fact, while the disruption of the lattice obviously hampers the proper running of the device, the distance between the graphene and the receptor is also a crucial factor for sensor functioning. In a solution-gated GFET, the gate is a reference electrode operating in an electrolyte solution. Under these conditions, the graphene surface is behaving as a polarisable electrode and the charges close to graphenes are organized in the classical double layer.^10^ Thus, the graphene suffers from the so-called Debye screening effect, namely, the capability of experiencing a charge carrier electric field only within the homonym Debye length (*λ*_D_). *λ*_D_ is strongly dependent on the solution ionic strength, being less than 1 nm under physiological conditions (PBS 10 mM).^24^ For this reason, the length of the linker used to immobilize the receptor onto the graphene must be controlled to obtain the best performances from the device.

**Fig. 2:**
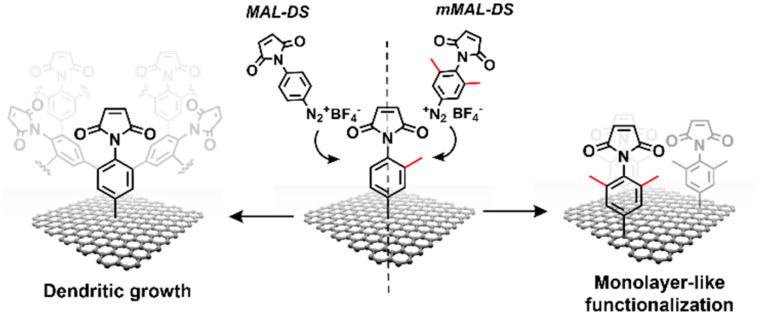
Schematic of the functionalisation using mMAL-DS (right) and MAL-DS (left) leading to monolayer-like and dendritic-like functionalisation, respectively.

**Fig. 3:**
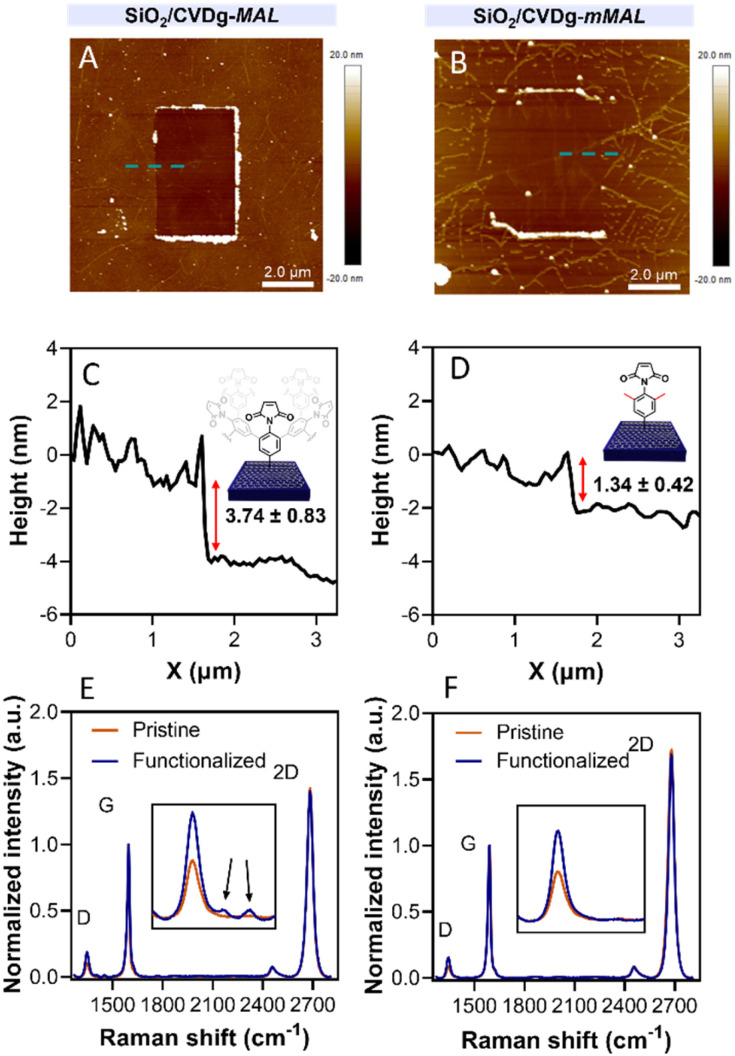
(A and B) AFM scratching images and (C and D) height profiles of SiO_2_/CVDg-MAL (A and C) and SiO_2_/CVDg-mMAL (B and D). (E and F) Raman spectra of SiO_2_/CVDg-MAL (E) and SiO_2_/CVDg-mMAL (F) before and after the reaction.

To avoid the dendritic growth of the aryl diazonium salt moieties and obtain monolayer-like functionalisation, we designed an aryl diazonium salt bearing two methyl groups in the *meta*-position to the diazonium. The utilisation of bulky groups in these positions was proved to be efficient to prevent the oligomerisation of the aryl diazonium salts.^23^ Additionally, our salt includes a maleimide group in the *para* position, employed for the aptamer conjugation by the thiol–maleimide Michael addition click reaction. To assess the efficiency of our molecular design in preventing the oligomerisation of the diazonium salt, we performed a comparative study of 4-(*N*-maleimido)-3,5-dimethylbenzenediazonium tetrafluoroborate salt (mMAL-DS) and 4-(*N*-maleimido)-benzenediazonium tetrafluoroborate salt (MAL-DS)-functionalised graphenes (Fig. 2). The reaction was performed on the graphene prepared by chemical vapour deposition and deposited on silicon oxide (SiO_2_/CVDg). The aryl diazonium salt solution (20 mM in water) was added dropwise to the SiO_2_/CVDg substrate soaked in water at r.t. It is worth noting that the occurrence of this spontaneous reaction could be attributed to impurities originating from the polymers employed in the transistor fabrication process, as well as the polar adsorbates on SiO_2_.^37^ Thus, in this electron transfer reaction, these substances facilitate the transfer of electrons from the occupied states of graphenes to the occupied states of the reacting species. After the reaction, the features of the functionalisation layer were evaluated by a combination of AFM and Raman spectroscopy analyses. First, by an AFM scratching experiment, we calculated the height of the functionalisation layer on the two substrates. This technique involves scratching the surface of the substrate with the AFM tip in a “contact mode” experimental setup, removing graphenes from the surface in a controlled way. By studying the height profile of the image and comparing the graphene with bare silicon oxide, the height of the functionalisation layer can be calculated. Indeed, since the height of the bare graphene is approximately 1.14 nm (Fig. S1†), we calculated the height of the two functionalised materials by subtraction, which resulted in heights of 0.20 nm and 2.58 nm for mMAL-DS-functionalised graphenes (SiO_2_/CVDg-mMAL) and MAL-DS-functionalised graphenes (SiO_2_/CVDg-MAL), respectively (Fig. 3A). This experimental evidence confirms the formation of a monolayer for the methylated salt and a multilayer for the unmethylated one. This result highlights the importance of the linker length for the sensing application. Indeed, as mentioned above, under physiological conditions, *λ*_D_ is almost 1 nm. Thus, the use of our molecularly designed mMAL-DS allows for anchoring the receptor within this distance, while the use of the unhindered MAL-DS would result in the receptor lying outside of the sensitive area of graphene transistors. Additional information could be obtained by performing AFM in the tapping mode. In previously published works,^23^ a remarkable change in the roughness (height distribution) of the substrate was associated with the dendritic growth of the functionalisation layer, while the monolayer-like functionalisation resulted in a retained roughness or minimal change.^2^ Therefore, the roughness of AFM images for pristine and functionalised samples using mMAL-DS, MAL-DS and 4-nitrobenzenediazonium tetrafluoroborate (NO_2_-DS) was compared. NO_2_-DS was chosen as a highly reactive aryl diazonium salt that is likely to form oligomers on the surface.^23,38^ The comparison presented in Fig. S2† showed that, while functionalisation with MAL-DS and NO_2_-DS induced a significant increase in the surface roughness compared to the pristine graphene, SiO_2_/CVDg-mMAL samples retained almost the same roughness. This result confirms our previous assumptions about oligomerisation.

Raman spectroscopy is a powerful technique in the study of chemical modification of graphenes. Usually, the increase in the D band with respect to the G band (*I*_D_/*I*_G_) is associated with the carbon hybridisation change from sp^2^ to sp^3^ within the lattice and is a common way to evaluate the material covalent functionalisation. In our samples, the increase in the D band suggests that the functionalisation degree of the two substrates is similar, with an *I*_D_/*I*_G_ ratio of 0.16 and 0.18 for SiO_2_/CVDg-mMAL and SiO_2_/CVDg-MAL, respectively (Fig. 3B). However, the Raman spectra of the SiO_2_/CVDg-MAL showed two extra peaks at 1400 and 1450 cm^−1^, typically obtained when using other “unprotected” salts, like the phenyl acetic diazonium salt (COOH-DS) or NO_2_-DS that are most likely forming oligomers during the functionalisation (Fig. S3†).^38^ The origin of these extra peaks is not completely understood nor extensively studied in the literature and our hypothesis is that it could be related to some stretching modes of the polyphenyl-like oligomers.^39^ Indeed, these peaks did not appear on the SiO_2_/CVDg-mMAL spectrum, where oligomerisation was prevented. The XPS analysis performed on the functionalised samples also corroborated the introduction of the maleimide groups by an increase in the N atomic percentage for both functionalisation (Fig. S4 and Table S1†). This outcome aligns with the previously reported findings.^40^ Furthermore, looking at the high-resolution C 1s spectra, an increase in the component associated with the N–C=O bond at 288.1 eV is noticed, related to the introduction of imides onto the surface.

### GFETs functionalisation

**Fig. 4:**
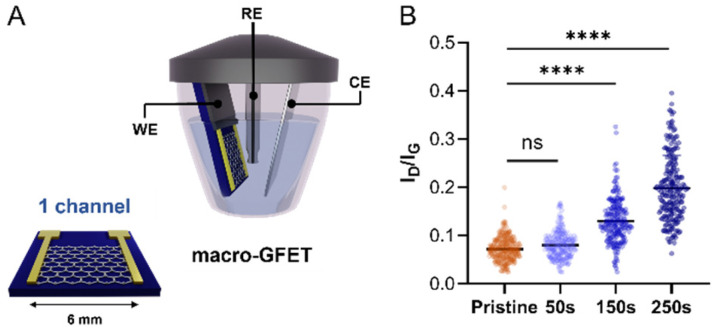
Electrochemical functionalisation of the macro-GFET. (A) Schematic of the macro-GFET and set up for the electrochemical reduction. (B) Ratio between the intensity of D band and the intensity of G band of the Raman spectra recorded on pristine (orange) and functionalized (shading blues) graphenes by applying increasing CA time.

Once confirmed that our molecular design enables the control over the functionalisation by avoiding the uncontrolled dendritic growth, mMAL-DS was employed for the GFET functionalisation. To gain additional control over the functionalisation process, an electrochemical diazonium salt reduction was chosen as the functionalisation strategy.^7,15,16^ This grafting methodology, often used in graphene device modification, resulted in controlled monolayer-like functionalisation when bulky groups are employed, inducing a reasonable increase in the lattice sp^3^ defects and almost no alteration in the 2D/G intensity (*I*_2D_/*I*_G_) ratio after careful optimisation.^23,28,41^ Therefore, the GFET was used as the working electrode shortcutting the source and drain electrodes in a three-electrodes configuration, employing Ag/AgCl (KCl 3 M) as the reference electrode (RE) and a platinum plate as the counter electrode (CE). The reaction was first optimized on individual GFETs (macro-GFETs) composed of a single channel of 6 × 6 mm^2^ area (Fig. 4A), in order to deeply characterize the functionalisation layer. The electrochemical functionalisation was performed by chronoamperometry (CA) which, compared to the widely used cyclic voltammetry (CV), provides the possibility of tuning the functionalisation degree by varying the potential application time. Indeed, even if the CV led to the grafting of aryl moieties on graphenes, the reduction is limited to the first scan (Fig. S5†), which results in a low functionalisation degree. However, when applying the reduction potential as a function of time, an increase in the D band intensity proportional to the reaction time is noticed (Fig. 4B and Fig. S6†). On macro-GFETs, a significant functionalisation is achieved after 150 s, which keeps increasing with the additional application time. Additionally, also after 250 s functionalisation, the transfer curve recorded on the device is not remarkably affected (Fig. S7†), confirming that the mildness of our controlled functionalisation can be used to produce functional devices. The XPS analysis confirmed the introduction of the maleimide group by an increase in the N atomic percentage from 0.7 to 2.3% (Fig. S8 and Table S2†). Furthermore, looking at the high-resolution C 1s spectra, an increase in the component associated with the N–C=O component at 288.1 eV is noticed, correlated with the introduction of imides onto the surface.

Certainly, the electrochemical method's control is highly adjustable, which allows reaching a degree of functionalization equivalent to that observed in the case of the free electrochemical strategy. After a comparative analysis of the data obtained by XPS and Raman spectroscopy in relation to electrochemical and free electrochemical modifications of graphenes, a consistent indication of similar functionalization is observed. XPS spectroscopy reveals congruent levels of N 1s content (a difference in the nitrogen content after functionalization, 1.2 (Table S1†) and 1.6 at% (Table S2†), for the electrochemical-free strategy and electrochemical reduction through CA for 250 seconds, respectively). Likewise, Raman spectroscopy demonstrates analogous manifestations of sp^3^ defects (*I*_D_/*I*_G_ is 0.16 (Fig. 3F) and 0.19 (Fig. S6A†), for the electrochemical-free strategy and electrochemical reduction through CA for 250 seconds, respectively).

To fully characterize our system, the number of available maleimide groups immobilized on the surface of GFETs was quantified by conjugation with a ferrocene redox mediator derivative bearing a terminal thiol. This methodology is widely used in the surface coverage study of different electrochemical systems.^28,42^ Indeed, the concentration of conjugated ferrocene can be calculated taking advantage of its electroactivity by performing CV on the functionalised electrode (Fig. S9†). For our system, we calculated a surface coverage of 2.6 × 10^−11^ mol cm^−2^, that is comparable to other reported functionalised GFETs.^25^

**Fig. 5:**
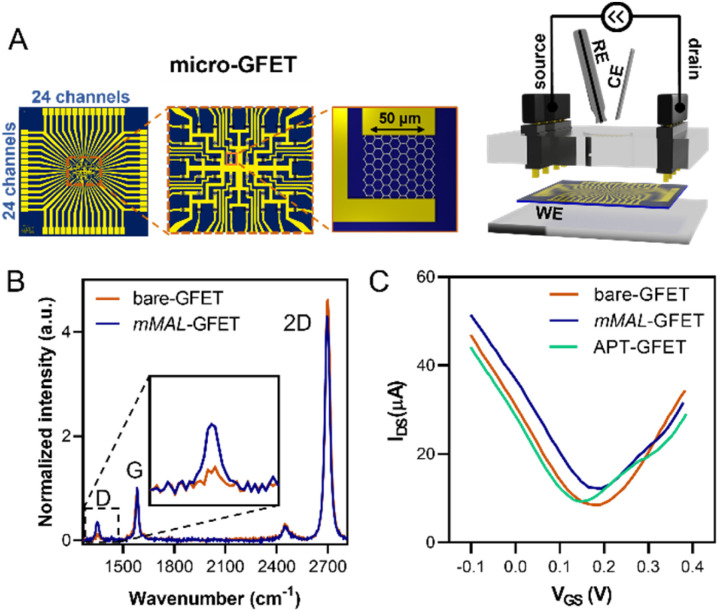
Micro-GFET electrochemical functionalisation characterisation. (A) Schematic of the micro-GFET device and electrochemical setup. (B) Raman spectra of the pristine bare-GFET and mMAL-GFET. (C) Transfer curves recorded on the micro-GFET recorded after each step of functionalisation.

Once corroborated our functionalisation on macro-GFETs, we translated it to the more complex system of the micro-GFET array, composed of 48 channels divided into two groups of 24 (Fig. 5A), in which graphene channels are 50 × 50 μm^2^ in area. The functionalisation set up is analogous to the one employed for macro-GFETs, with 24 channels of the micro-GFET connected as the working electrode in a three-electrode configuration. For this system, we found that by applying a reduction potential of −0.5 V *vs.* Ag/AgCl (KCl 3 M) for 100 seconds, a good compromise between the functionalisation degree and electronic property preservation is achieved. After grafting, the transfer curve recorded on the functionalised device (mMAL-GFET) resulted in a slight right shift due to p-doping typical of aryl diazonium grafting (Fig. 5C),^25,28^ with no remarkable changes in the mobility of graphenes (Fig. S10†). The maleimide group introduced on the graphene surface of the micro-GFET obtained by electrochemical functionalisation was subsequently utilized to conjugate the serotonin stem-loop aptamer. The conjugation process involved the typical reaction between the thiol moiety introduced at the 5′ ends of the aptamer chain and the maleimide on the surface. Thus, the aptamer was pre-treated with a tris(2-carboxyethyl)phosphine (TCEP) reducing agent to ensure the availability of free thiols. Additionally, before the incubation, the aptamer was refolded by 10 minutes heating at 90 °C followed by cooling at r.t. The aptamer was then diluted to a 1 μM final concentration in 10 mM PBS and incubated on the graphene surface overnight. The unreacted maleimide sites remaining after the incubation were blocked using a 2-mercaptoethanol solution to ensure that no reactive species is left on the surface. The transfer curves recorded after the conjugation and blocking (APT-GFETs) resulted in a negative shift of the Dirac point (Fig. 5C). As previously described, the transport characteristics of graphene in a solution-gated GFET are led by the graphene/electrolyte interface. In the case of aptamer conjugation, predicting the doping sense becomes complicated due to the adsorption of DNA and counter ions on graphenes, as well as the morphology of DNA chain. In particular, the n-doping registered in our case is typical of single-stranded DNA chains. This is because the non-hybridized nucleosides freely interact with graphenes, competing with the phosphate group for doping.^43^ Additionally, in the case of single-stranded DNA possessing a hairpin loop, such as in our study, the interaction of the nucleosides prevails, resulting in a left shift of the transfer curve, as observed in most reported cases.^25,34,43^ Moreover, in the case of aptamer conjugation, the modification does not alter the electronic properties of the micro-GFET.

### Serotonin real-time monitoring

The serotonin monitoring experiments involve the real-time recording of the changes induced on graphene transistors by the injection of serotonin of different concentrations. To better simulate real-time monitoring, we performed the experiment using a polymethyl methacrylate (PMMA) flow cell (Fig. S11†) with a peristaltic flow pump to control the flow rate.

**Fig. 6:**
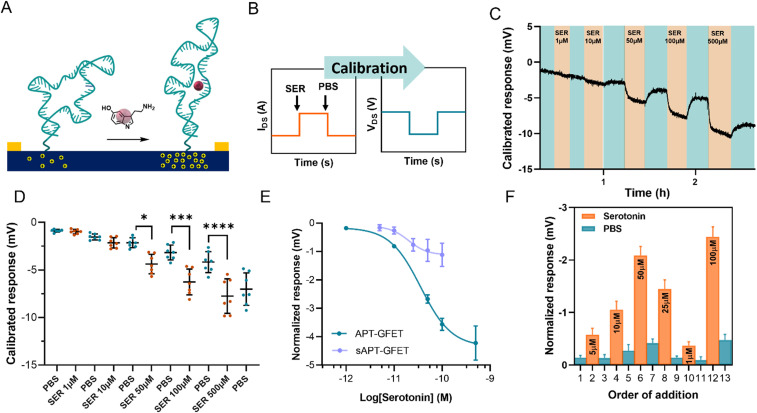
(A) Schematic of the aptamer conformational change upon recognition of serotonin. (B) Current *vs.* time recording during real-time monitoring and subsequent calibration to the final data of voltage *vs.* time. (C) Calibrated voltage response of different channels upon the addition of serotonin at different concentrations alternated with 10 mM PBS injections. (D) Dot plot of the calibrated voltage response of different channels at selected timepoints. The response is expressed as average ± SD, *n* = 7. One-way ANOVA statistical analysis (*P < 0.05, ***P < 0.0005, ****P < 0.0001). (E) Normalized response of the APT-GFET and sAPT-GFET to different concentrations of serotonin. The serotonin concentration is expressed in log10 scale to obtain a sigmoidal fit. The response is expressed as average ± SD, *n* = 7. (F) Calibrated voltage response upon the addition of serotonin at random concentrations. The response is expressed as average ± SD, *n* = 7.

During the measurements, the device current was constantly recorded while changing the flowing solution. The experiment involved recording the current between the source and the drain (*I*_DS_) *vs.* time at a fixed polarisation voltage (*V*_GS_). For our system, we selected a potential of 0 V *vs.* Ag/AgCl (KCl 3 M) since the transfer curve exhibited greater reliability and stability in the hole domain as opposed to the electron domain. The calibration was carried out by injecting serotonin at a concentration ranging between 1 μM and 500 μM in 10 mM PBS alternated with PBS injections, using a flow injector. Upon addition of serotonin, an increase in current at *V*_GS_ = 0 V was recorded. This increment in current is associated with a positive shift of the transfer curve, indicating p-doping caused by the aptamer conformational change. This behaviour is in accordance with the described recognition mechanism, where the binding of the analyte straightens the folded aptamer and drives it away from the graphene surface (Fig. 6A).^30^ Since the introduction of the aptamer induced a negative shift to the Dirac point of micro-GFETs, the recognition event was characterized by a positive shift because of the straightening of the oligonucleotide. The recorded signal is then calibrated by employing the micro-GFET channel transfer curve recorded before the time plot to pass from current (*I*_DS_) *vs.* time to voltage (*V*_GS_) *vs.* time (Fig. 6B). This calibration procedure is convenient, as it helps to eliminate the variations associated with different current levels and resistance for each channel.

The calibrated response of a micro-GFET array is included in Fig. 6C (and Fig. S12†). Here, the response given by the subsequent addition of serotonin at different concentrations alternated with 10 mM PBS injection evidenced the reversible nature of the recognition system. This reversibility of the binding is possible thanks to the labile interaction between the receptor and the serotonin, with a reported dissociation constant of 30 nM in solutions.^30^ From the calibrated time plot (*I*_DS_*vs.* time), we extracted the voltage values corresponding at the end of the plateau for each addition before injecting the next solution. Thus, analysing all the channels, the dot plot presented in Fig. 6D was constructed. This result demonstrates the power of working with the micro-GFET array, where a statistically significant result is obtained by a single experiment. Due to inhomogeneity in the fabrication characteristic and current drift of the different channels, the response has a wide SD, affecting the quality of the measure. Additionally, it is worth mentioning that SD remarkably increased after the addition of the higher concentration of serotonin. This suggests that not complete reversibility is produced after saturation.

The signal from each GFET was then normalized by subtracting the PBS signal intensity recorded before each addition to construct the calibration curve (Fig. 6E). The calibration exhibited a typical binding-to-saturation behaviour that was fit using a sigmoid function. The signal is proportional to the concentration of serotonin, reaching a plateau at 500 μM and is reversed by the addition of 10 mM PBS. The sensitivity (*S*) of the sensor, which is defined as Δ*I*_DS_/Δ*V*_GS_, was calculated as the slope of the linear regression of the linear portion of the calibration curve (1–100 μM). This value was employed, along with the noise (*σ*, standard deviation of the blank), to calculate the limit of detection (LOD), which resulted to be of 11.4 μM (see ESI†). To confirm that the recorded signal is attributed to the aptamer–serotonin interaction, we conducted a control experiment by modifying a micro-GFET array with a scrambled sequence of the serotonin aptamer (sAPT-GFET). This scrambled sequence has already been tested and described to have low serotonin recognition efficiency.^30,32^ As shown in Fig. 6E, the signal produced by the scrambled sequence interaction is negligible compared to the correct sequence, thus confirming the recognition conformational change mechanism. To additionally assess the reversibility and stability of our system, we performed a random addition experiment. Here, we added the serotonin solution in a random order using the same setup and operating method of the standard calibration, instead of an increasing manner as previously done (Fig. 6F). Our sensor demonstrated the ability to record signals proportional to the concentration in a random manner when operating below the saturation limit (500 μM). This confirms the recovery of the receptor's starting conformation upon the addition of PBS after recognition.

## 3. Conclusions

Our work demonstrated the possibility of efficiently employing covalent functionalisation of graphenes based on aryl diazonium salts to develop a functional GFET biosensor. By means of molecular design and electrochemical triggering, our strategy gives the opportunity of finely controlling the functionalisation degree to obtain a good compromise between the functionalisation degree and lattice disruption for each application. In the present work, we employed the methodology to introduce an organic linker bearing a maleimide group on the surface of graphene micro-GFET arrays. The maleimide was then used to conjugate a serotonin stem-loop aptamer on the device surface by thiol–maleimide Michael-type addition. The device selectively recognized serotonin in a reversible manner in the range between 10 and 500 μM with a calculated LOD of 11.4 μM. Remarkably, our device works in high-ionic strength solutions (10 mM PBS, *λ*_D_ = 0.74 nm), which have comparable salinity to physiological fluids such as cerebrospinal fluid (*λ*_D_ = 0.76 nm calculated for artificial cerebrospinal fluid; Table S5†). This confirmed the possibility of overcoming the limitation of the Debye screening effect through the linker molecular design. This proof of concept paves the way for the development of new platforms for neurotransmitter monitoring thanks to the great stability of the covalently attached linker.

## Author contributions

Cecilia Wetzl: conceptualisation, investigation, methodology, formal analysis and writing – original draft. Sergi Brosel-Oliu: investigation, methodology, formal analysis and writing – review & editing. Desiré Di Silvio: investigation, methodology and writing – review & editing. Marco Carini: investigation, methodology and writing – review & editing. Xavi Illa: sample manufacture, validation and investigation. Rosa Villa: supervision, project administration, funding acquisition and writing. Anton Guimera: supervision, methodology and writing – review & editing. Elisabet Prats-Alfonso: conceptualisation, investigation, writing – review & editing. Maurizio Prato: supervision, project administration, funding acquisition and writing – review & editing. Alejandro Criado: conceptualisation, methodology, supervision, project administration, funding acquisition and writing – review & editing.

## Conflicts of interest

There are no conflicts to declare.

## Supplementary Material

NR-015-D3NR04153K-s001
