# Supplementary material for: Covalent functionalisation controlled by molecular design for the aptameric recognition of serotonin in graphene-based field-effect transistors
Source: Nanoscale. 2023 Sep 19;15(41):16650–7. doi: 10.1039/d3nr04153k (PMC10600654; doi:10.1039/d3nr04153k)
Supplement: NR-015-D3NR04153K-s001 [file NR-015-D3NR04153K-s001.pdf]

Electronic Supplementary Material (ESI) for Nanoscale.
This journal is © The Royal Society of Chemistry 2023

Supporting information

Covalent functionalisation controlled by molecular design for aptameric recognition of serotonin in graphene-based field-effect transistors

Cecilia Wetzl[a,b], Sergi Brosel-Oliu[c], Marco Carini[a], Desiré Di Silvio[a], Xavi Illa[c,d], Rosa Villa[d,c], Anton Guimera-Brunet[d,c], Elisabet Prats-Alfonso[c,d,*], and Maurizio Prato[a,e,f,*], Alejandro Criado[g,a,*]

[a] *Center for Cooperative Research in Biomaterials (CIC biomaGUNE), Basque Research and Technology Alliance (BRTA), Paseo de Miramón 194, 20014, Donostia-San Sebastián, Spain. E-mail: mprato@cicbiomagune.es*

[b] *University of the Basque Country, UPV-EHU, 20018 San Sebastián, Spain*

[c] *Centro de Investigación Biomédica en Red de Bioingeniería, Biomateriales y Nanomedicina, Instituto de Salud Carlos III).*

[d] *Instituto de Microelectrónica de Barcelona, IMB-CNM (CSIC), Campus UAB, Bellaterra, Spain. Email: elisabet.prats@csic.es*

[e] *Ikerbasque, Basque Foundation for Science, Bilbao, Spain.*

[f] *Dipartimento di Scienze Chimiche e Farmaceutiche, Università degli Studi di Trieste, Trieste, Italy.*

[g] *Universidade da Coruña, CICA – Centro Interdisciplinar de Química e Bioloxía, Rúa as Carballeiras, 15071 A Coruña, Spain. E-mail: a.criado@udc.es*

Table of contents

Material and methods 3
Synthesis of *mMAL-DS* 6
Synthesis of *MAL-DS* 9
Synthesis of *NO_2-DS* 10
Synthesis of *COOH-DS* 11
GFET fabrication 11
Figure S1. AFM scratching SiO_2/CVDg 12
Figure S2. AFM micrographs of SiO_2/CVDg functionalized samples 12
Figure S3. Raman spectra of SiO_2/CVDg functionalized samples 13
Figure S4. XPS characterisation of SiO_2/CVDg-*mMAL* and SiO_2/CVDg-*MAL*. 13
Table S1. XPS analysis of SiO_2/CVDg-*mMAL* and SiO_2/CVDg-*MAL* 14
Figure S5. Characterisation of macro-GFET functionalized by CV. 14
Figure S6. Raman characterisation of mMAL-macro-GFET 15
Figure S7. Transfer curve of macro-GFET. 15
Figure S10. Charge carrier mobility of micro-GFET. 17
Figure S11. Flow cell used for the micro-GFET calibration experiments. 18
Figure S12. Calibrated response to serotonin addition on GFETs array. 19
Debye length calculation 19
Limit of detection 19

Material and methods

All the chemicals for the synthesis of the mMAL-DS and MAL-DS were purchased from Sigma-Aldrich, Germany. Phosphate buffer saline tablets (PBS), potassium nitrate (KNO3, ≥99.0%), tris(2-carboxyethyl)phosphine hydrochloride (TCEP, ≥98.0%), serotonin hydrochloride (≥98%) and 2-mercapto ethanol (55 mM in PBS) were also from Sigma-Aldrich. Zeba™ Spin Desalting Columns, 7 K MWCO, 0.5 mL were purchased from Thermofisher, France. The 5'-thiol modified aptamer (5'-CGA CTG GTA GGC AGA TAG GGG AAG CTG ATT CGA TGC GTG GGT CG-3') and scrambled sequence (5'-CCC GGG AAT TCC GGA ATT GGG GCA ATT GAT GAG GGG GTC ATG GG-3') were purchased from Microsynth, Switzerland. Chemical vapour deposited graphene (CVDg) on copper was purchased from Graphenea, Spain.

AFM micrographs were registered using a JPK BioAFM microscope (Bruker Nano GmbH), employing a tapping mode tip with the frequency of 320 Hz (Bruker TESPA-V2). Shaving experiments were performed on a Bruker Multimode 8 microscope in Contact mode scanning several times an area of 10 µm x4 µm and applying a force of ≈70 nN at a scan rate of 2Hz. The probe used was the ScanAsyst-Air silicon tip (Bruker). The shaved area was characterized again in PeakForce QNM mode with a scan angle of 90o to avoid artifacts do to piled material. AFM images were processed using WSvM5.0 and Nanoscope Analysis 2.0 software. XPS measurements were performed in a SPECS Sage HR 100 spectrometer using a non-monochromatic X-ray source of Mg with a Ka line of 1253.6 eV. An electron flood gun was used to avoid sample charging. XPS data fitting was performed using Casa XPS software (Version 2.3.16 PR 1.6). Raman spectra were recorded using a Renishaw Invia Raman spectrometer (lex = 532 nm). Each spectrum is the average of at least 300 spectra recorded in different spots of the sample with 1 s of integration time at a laser power of 1.29 mW. Data were processed using Ranishaw WiRE 4 software. All NMR data were collected on a Bruker AVANCE III NMR spectrometer (11.7 T, 500 MHz for 1H). The spectra were processed with MestReNova software version 7.1.1-9649. Electrochemical characterizations were performed through CV and at constant potential with an electrochemical workstation Autolab MSTAT204 potentiostat/galvanostat (Metrohm) and a Solatron Analytical 1287A Potentiostat Galvanostat.

SiO_2/CVDg thermal decomposition functionalization A solution of 4-(N-maleimido)-3,5-dimethylbenzenediazonium tetrafluoroborate salt (mMAL-DS) or 4-(N-maleimido)-benzenediazonium tetrafluoroborate salt (MAL-DS) (20 mM in Milli Q water, 2 mL) was added dropwise to the SiO_2/CVDg soaked in Milli Q water (10 mL) using a syringe pump system (flow rate 2.5 mL min^{-1}). The substrate was then rinsed with abundant Milli Q water and ethanol and dried with N_2 flow.

Graphene CVD growth and transfers Graphene was grown by Chemical Vapor Deposition (CVD) method. Black magic Pro 4" CVD Aixtron Reactor and 25 µm thick, 99.8% metal basis copper foil

provided by Alfa Aesar have been employed. Prior to CVD process, copper foils were cut in 6 x 5 cm^2 samples and sequentially cleaned in acetic acid and acetone, and finally rinsed in isopropyl alcohol (IPA). Graphene growth process was composed by two steps; i) 10 minutes Cu annealing at 1000 °C, with flow of 400 standard cubic centimetres per minute (sccm) of H_2, 600 sccm of Ar and 150 sccm of N_2, followed by ii) 10 minutes graphene growth at 970 °C using methane as carbon precursor (10/100 sccm CH_4/ H_2 ratio). A 700 nm thick sacrificial layer of polymethyl methacrylate (PMMA, 7% 950k MW PMMA dissolved in anisole, provided by Micro Resist technology GmbH, DE) was deposited by spin-coating above graphene. CVD graphene was delaminated from the Cu foil by the electrochemical method.[42] Before the transfer process, supporting substrates were activated by UVO cleaner for 5 minutes. After transfer, samples underwent to a 40 min temperature ramp from r.t. to 180°C and a final bake at 180 °C for 2 minutes. After cooling to r.t., the PMMA sacrificial layer was removed by immersing samples in acetone and then in isopropanol, for 30 minutes in each solvent.

GFET fabrication The same fabrication process was used to manufacture the macro-GFET and the 14-channel single-address GFET and the 48-channel micro-GFET. A conventional lift-off process using the image reversal photoresist AZ5214E (Clariant, Germany) is followed in four-inch silicon wafers covered by 2 µm thick thermal oxide. The bottom metal layer is an e-beam evaporated Ti/Au, 10/100 nm in thickness. After graphene transfer, the graphene GFET active areas were defined by means of an oxygen-based reactive ion etching using a patterned HiPR 6512 resist mask which is subsequently removed in acetone. The patterning process of the top metal layer is similar to the bottom metal contact patterning, but the metal stack consisted in a Ni/Au, 20/200 nm thick film. Finally, SU-8 negative photoresist (SU-8 2005, MicroChem, USA) is used to passivate the metal leads while defining the graphene channel and metal contacts openings.

GFET functionalization The electrochemical functionalization was performed using the macro/micro-GFET as WE in a tree-electrode configuration using Ag/AgCl (KCl 3M) as a RE and platinum plate as a CE. The electrodes were soaked in a solution mMAL-DS in 50% ethanol with 0.1 M KNO_3 (5 mM) as support electrolyte. The functionalization was produced by performing a CA at -0.5 V vs Ag/AgCl (KCl 3M) for 100s (unless stated otherwise). The GFET was then rinsed with ethanol and Milli Q water and dried. The electrografting performed by CV (E= -0.7: 0 V vs Ag/AgCl (KCl 3M), 3 or 4 scans, 0.1 V s^{-1} scan rate) was performed with the same experimental set up and mMAL-DS concentration.

Ferrocene-SH conjugation 50 µL of tris(2-carboxyethyl)phosphine (TCEP) solution (10 mM in PBS 10 mM) was freshly prepared and mixed with of PBS (10 mM, 100 µL) and Ferrocene-C_6H_{12}-SH solution (2 mM in EtOH, 50 µL). The mixture was shaken for 30 minutes and then the solution (100 µL) was incubated overnight over the device. The device was then washed with H_2O and EtOH before further analysis.

Stem-loop aptamer conjugation The as received aptamer was diluted up to 100 µM in 10 mM PBS containing 50-fold excess of TCEP and incubated for 1 hour. The solution was purified using a ZEPA® desalting column (MWCO 7kDa) before aliquoting and storing at -20°C until further use. Prior the surface conjugation, the aptamer was refolded by treatment at 90 °C for 10 minutes followed by cooling at r.t.. The solution was then diluted to 1 µM in 10mM PBS and incubated on the device overnight. The device was then delicately rinsed with Milli Q water and gently dried. The surface groups were subsequently blocked by incubating a solution 2-mercapto ethanol (1 mM in 10 mM PBS) for 20 minutes. The device was washed and dried prior to further characterization.

Electrical characterization of GFET The setup to perform the measurements is composed by a Printed Circuit Board (PCB) connected to a Data Acquisition Card (DAQ Card), which digitalizes and transmits to a computer the analogue signal and allow the measurement of 24 transistors of each probe and the Direct Current (DC) source that polarize the operational amplifiers from the PCB. The transfer curves were recorded in 10mM PBS sweeping the V_{GS} between -0.1 and 0.4 V using an Ag/AgCl (KCl 3M) RE as the gate. The device was connected to the electrical setup through a PMMA cell equipped with gold arrays in touch with the gold contacts. The V_{DS} was maintained fixed to 0.05 V vs Ag/AgCl (KCl 3M) for all the experiments.

Serotonin monitoring A PMMA flow cell connected to a syringe pump through a 6-port valve. was connected to the Ag/AgCl (KCl 3M) gate by the outlet tube ending in a 10 mM PBS, i.e. PBS 1x, reservoir. Here, the V_{GS} was fixed at 0 V vs Ag/AgCl (KCl 3M). During the experiment the flow rate was kept at 100 µL min^{-1} during the injection. Then it was lowered to 10 µL min^{-1} once the solution reached the cell and maintained for 8 min, before injecting the following solution. The data were analysed via a Python 3 scripts.

Synthesis of *mMAL-DS*

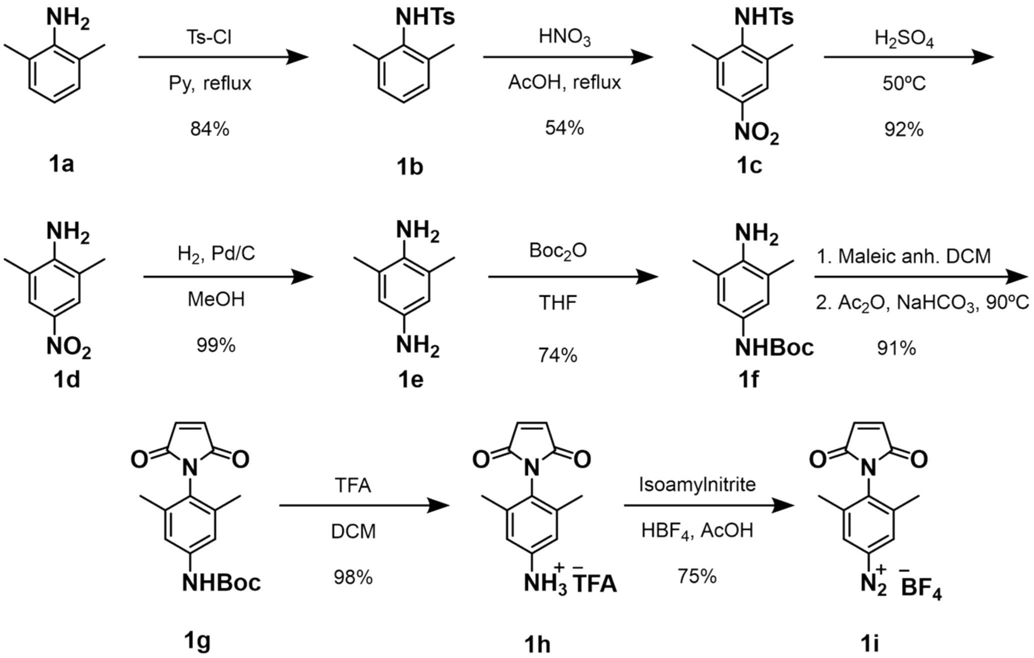

Scheme 1. Synthetic route for *mMAL-DS* (1i).

2',6'-dimethyl-p-toluensulfanilide (1b) was synthetized according to a reported procedure. 2,6-dimethylaniline (82.5 mmol, 10.0 g) and tosyl chloride (123.7 mmol, 23.6 g) were refluxed in pyridine (40 mL) for 30 minutes. The reaction mixture was poured on crushed ice, the precipitate was filtered and washed with water and MeOH. The compound (69.3 mmol, 19.04 g) was obtained as a white solid in 84% yield.

^{1}H NMR (500 MHz, Chloroform-d) δ: 7.59 (d, J = 8.3 Hz, 2H), 7.24 (d, J = 8.1 Hz, 2H), 7.09 (dd, J = 8.2, 6.8 Hz, 1H), 7.01 (d, J = 7.5 Hz, 2H), 5.92 (bs, 1H, NH), 2.42 (s, 3H), 2.04 (s, 6H) ppm.

2',6'-dimethyl-4-nitro-p-toluensulfanilide (1c) was synthetized according to a modified literature procedure. 2',6'-dimethyl-p-toluensulfanilide (67.4 mmol, 18.6 g) was dissolved in ethyl acetate (50 mL) at reflux. 99% HNO_3 (74.1 mmol, 3.0 mL) was dissolved in ethyl acetate (20 mL) were added dropwise for 10 minutes and the reflux was continued for 2h30'. The reaction mixture was poured on crushed ice, the precipitate was filtered and washed with water. The solid was redissolved in dichloromethane, activated charcoal was added and the mixture was stirred for 20 minutes and filtered through celite. The solvent was evaporated, and the product was finally triturated with methanol. The compound **1c** (36.4 mmol, 11.91 g) was obtained as a white solid in 54% yield.

^{1}H NMR (500 MHz, Chloroform-d) δ: 7.89 (s, 2H), 7.60 (d, J = 8.3 Hz, 2H), 7.29 (d, J = 7.9 Hz, 1H), 6.14 (bs, 1H, NH), 2.45 (s, 3H), 2.15 (s, 6H) ppm.

2,6-dimethyl-4-nitroaniline (1d) was synthetized according to a reported procedure. 2',6'-dimethyl-4-nitro-p-toluensulfanilide (37.2 mmol, 11.9 g) was dissolved in 96% H_2SO_4 (60 mL) and stirred at

50ºC for 1h30'. The reaction mixture was poured on crushed ice, the pH was neutralized with NH_4OH and the precipitate was filtered and washed with water. The compound (32.0 mmol, 5.46 g) was obtained as an ochre-yellow solid in 88% yield.

^1H NMR (500 MHz, Chloroform-d) δ: 7.90 (s, 2H), 4.26 (bs, 2H, NH_2), 2.23 (s, 6H) ppm.

2,6-dimethyl-1,4-diaminobenzene (1e). 2,6-dimethyl-4-nitroaniline (6.1 mmol, 1.02 g) was dissolved in methanol (100 mL), the solution was deoxygenated by bubbling Argon through the mixture for 10 minutes, then Pd/C was added (10% mol, 0.6 mmol, 64 mg). The Argon atmosphere was replaced with H_2 by means of 3 vacuum-H_2 cycles and the reaction was stirred overnight. The reaction was filtered on celite, and the solvent was evaporated. The compound (6.0 mmol, 825 mg) was obtained as a violet waxy solid in 99% yield.

^1H NMR (500 MHz, Chloroform-d) δ: 6.39 (s, 2H), 3.23 (bs, 4H, 2xNH_2), 2.13 (s, 6H) ppm.

tert-butyl N-(4-amino-3,5-dimethylphenyl) carbamate (1f) was synthetized according to a reported procedure. 2,6-dimethyl-1,4-diaminobenzene (8.9 mmol, 1.21 g) was dissolved in THF (100 mL) at 0ºC under Argon atmosphere. Boc_2O (8.9 mmol, 1.94 g) was dissolved in THF (50 mL) and added dropwise for 30 minutes. The reaction was allowed to warm to rt and it was stirred overnight. The solvent was evaporated under reduced pressure and the product was purified by column chromatography in hexane / AcOEt (80:20 → 70:30). The product was decanted in hexane (20 mL), the coloured supernatant was removed, and the precipitate was washed with hexane (2 x 5 mL). The compound (8.8 mmol, 1.56 g) was obtained as a pale violet solid in 99% yield.

^1H NMR (500 MHz, Chloroform-d) δ: 6.94 (s, 2H), 6.19 (bs, 1H, NH), 3.43 (bs, 2H, NH_2), 2.16 (s, 6H), 1.50 (s, 9H) ppm.

N-(4-tert-butoxycarbamoyl-2,6-dimethylphenyl)maleimide (1g). tert-butyl N-(4-amino-3,5-dimethylphenyl) carbamate (6.5 mmol, 1.54 g) was dissolved in dichloromethane (15 mL) and of maleic anhydride (7.1 mmol, 705 mg) were added and the rection was stirred overnight. The solvent was evaporated under reduced pressure and the residue was resuspended in acetic anhydride (30 mL) with of $NaHCO_3$ (7.2 mmol, 604 mg) and the reaction was stirred at 90ºC for 4h30'. The mixture was cooled to r.t. and quenched with water and saturated $NaHCO_3$ solution. The product was extracted with Et_2O and purified by column chromatography in hexane / AcOEt (70:30). The compound (5.9 mmol, 1.87 g) was obtained as a pale-yellow solid in 91% yield.

^1H NMR (500 MHz, Chloroform-d) δ: 7.17 (s, 2H), 6.85 (s, 2H), 6.50 (bs, 1H, NH), 2.06 (s, 6H), 1.51 (s, 9H) ppm.

^{13}C NMR (126 MHz, Chloroform-d) δ: 169.84, 152.63, 139.32, 137.95, 134.44, 123.82, 118.07, 80.88, 28.44, 18.24 ppm.

N-(4-amino-2,6-dimethylphenyl)maleimide TFA salt (1h). N-(4-tert-butoxycarbamoyl-2,6-dimethylphenyl)maleimide (5.9 mmol, 1.87 g) was dissolved in dichloromethane (10 mL) and trifluoroacetic acid (10 mL) and the reaction was stirred for 2h30'. The solvent was evaporated under reduced pressure and the residue was co-evaporated with Et_2O, methanol, toluene and Et_2O until as solid was obtained. The product was finally triturated with Et_2O / hexane, filtered and washed with hexane. The compound (5.6 mmol, 1.88 g) was obtained as a cream-white solid in 96% yield.

^{1}H NMR (500 MHz, DMSO-d6) δ: 7.84 (bs, 3H), 7.21 (s, 2H), 6.63 (s, 2H), 1.92 (s, 6H) ppm.

^{13}C NMR (126 MHz, DMSO-d6) δ: 170.21, 137.62, 134.7, 116.55, 17.52 ppm.

4-(N-maleimido)-3,5-dimethylbenzenediazonium tetrafluoroborate (1i, *mMAL-DS*). N-(4-amino-2,6-dimethylphenyl)maleimide TFA salt (1.4 mmol, 463 mg) and 48% v/v HBF_4 (500 µL) were dissolved in acetic acid (98%, 10 mL). Isoamylnitrite (500 µL) was dissolved in acetic acid (5 mL) and added dropwise to the mixture. After 20 minutes Et_2O (10 mL) were added slowly until some turbidity appears. The mixture was cooled to -20ºC for 1h30' and the resulting precipitate was collected by filtration and washed with Et_2O. The compound (1.05 mmol, 332 mg) was obtained as a white solid in 75% yield.

^{1}H NMR (500 MHz, Acetonitrile-d3) δ: 8.30 (s, 2H), 7.08 (s, 2H), 2.29 (s, 6H) ppm.

^{13}C NMR (126 MHz, Dimethylsulfoxide-d6) δ: 170. 08, 137.35, 134.53, 115.97, 17.40 ppm.

^{19}F NMR (471 MHz, Acetonitrile-d3) δ: -151.69, -151.74 ppm.

^{1}H NMR of *mMAL-DS* in acetonitrile-d3.

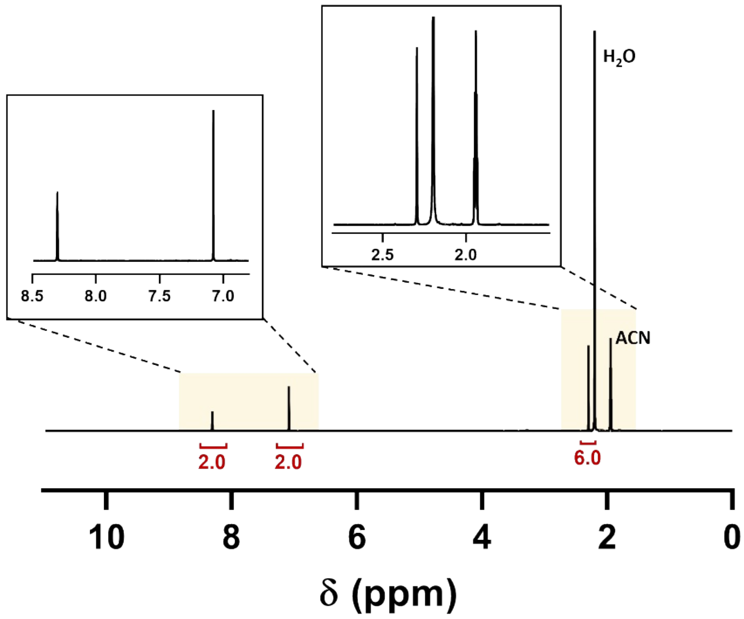

^{13}C NMR of *mMAL-DS* in Dimethylsulfoxide-d6.

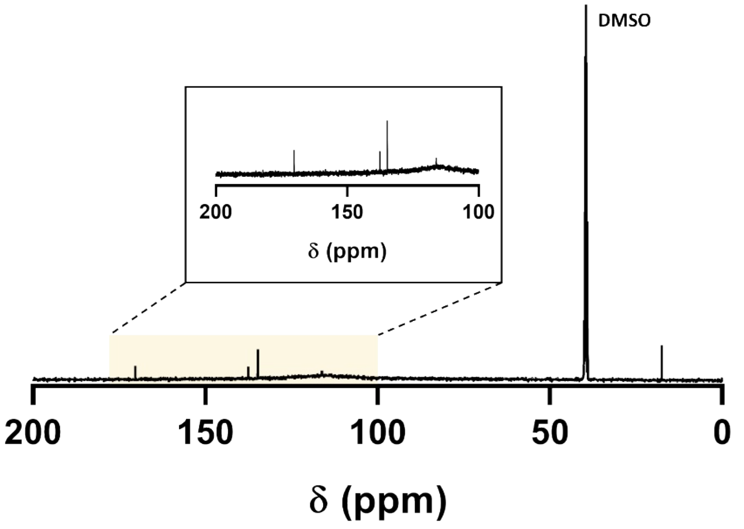

^{19}F NMR of *mMAL-DS* in acetonitrile-d3.

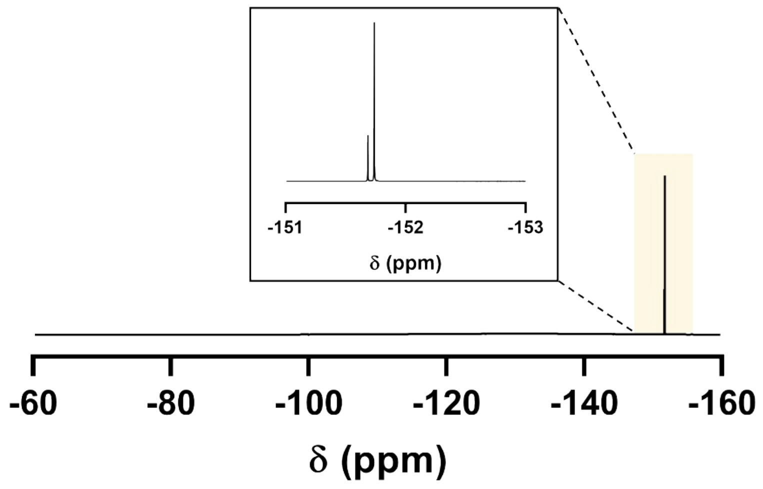

Synthesis of *MAL-DS*

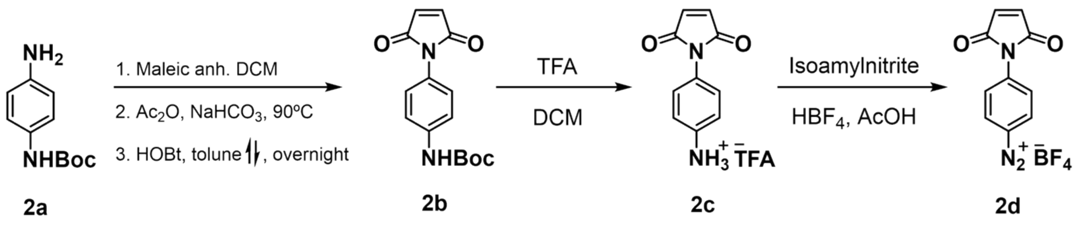

Scheme 2. Synthetic route for *MAL-DS* (2d).

N-(4-tert-butoxycarbamoyl phenyl)maleimide (2a). tert-butyl N-(4-amino phenyl) carbamate (5 mmol, 1.04 g) was dissolved in dichloromethane (20 mL) and maleic anhydride (5 mmol, 490.3 mg) in dichloromethane (5 mL) was added. The reaction was stirred for 1h and then the mixture was

filtered and washed with EtOH. The product was obtained as a green solid (4.15 mmol, 1.273 g, Y=83%). The product (2.4 mmol, 750 mg) was resuspended in acetic anhydride (35 mL) with $NaHCO_3$ (2.9 mmol, 240 mg) and the reaction was stirred at 90 ºC for 30'. The mixture was cooled to r.t., quenched with water, extracted with dichloromethane (2x 150 mL) and washed with saturated $NaHCO_3$ solution (100 mL). The product was purified by column chromatography in dichloromethane. The yellow solid obtained is a mixture of maleimide and isomaleimide. The obtained product (1.4 mmol, 435 mg) was converted to pure maleimide dissolving the solid in toluene and adding hydroxybenzotriazole (HOBt, 10% mol, 0.14 mmol, 20 mg). The mixture was then refluxed overnight. The crude was purified by column chromatography in 100% dichloromethane. The compound (1.1 mmol, 307 mg) was obtained as an orange solid in 46% yield.

^{1}H NMR (500 MHz, Chloroform-d) δ: 7.17 (s, 2H), 7.07 (d, 2H), 6.85 (d, 2H), 6.56 (bs, 1H, NH), 1.55 (s, 9H) ppm.

N-(4-amino phenyl)maleimide TFA salt (2b). N-(4-tert-butoxycarbamoyl phenyl)maleimide (0.74 mmol, 213 mg) was dissolved in dichloromethane (4 mL) and trifluoroacetic acid (4 mL) and the reaction was stirred for 2h. The solvent was evaporated under reduced pressure, and the residue was co-evaporated with Et_2O, MeOH, toluene and Et_2O until a solid was obtained. The product was finally triturated with Et_2O / hexane, filtered, and washed with hexane. The compound (0.71 mmol, 216 mg) was obtained as an orange solid in a 96% yield.

^{1}H NMR (500 MHz, DMSO-d6) δ: 7.17 (s, 2H), 7.07 (d, 2H), 6.85 (d, 2H) ppm.

4-(N-maleimido) benzenediazonium tetrafluoroborate (2c). N-(4-amino-2,6-dimethylphenyl)maleimide TFA salt (1.12 mmol, 213 mg) and 48% v/v HBF_4 (500 μL) were dissolved in acetic acid (10 mL), isoamylnitrite (500 μL) was dissolved in acetic acid (5 mL) and was added dropwise. After 20 minutes , Et_2O (10 mL) were added slowly until some turbidity appears. The mixture was cooled to -20ºC for 1h30' and the resulting precipitate was collected by filtration and washed with Et_2O. The compound (0.87 mmol, 158 mg) was obtained as a white solid in 78% yield.

^{1}H NMR (500 MHz, Acetonitrile-d3) δ: 8.58 (d, 2H), 8.16 (d, 2H), 7.11 (s, 2H), ppm.

Synthesis of *NO_2-DS*

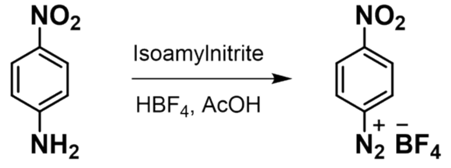

3a 3b

Scheme 3. Synthetic route for *NO_2-DS* (3b).

4-nitrobenzenediazonium tetrafluoroborate (3b). 4-nitrobenzene amine (5 mmol, 690 mg) and 48% v/v HBF_4 (2 mL) were dissolved in acetic acid (40 mL), isoamylnitrite (2 mL) was dissolved in acetic acid (20 mL) and was added dropwise. After 20 minutes Et_2O (10 mL) were added slowly until some turbidity appears. The mixture was cooled to -20ºC for 1h30' and the resulting precipitate was collected by filtration and washed with Et_2O. The compound (3.4 mmol, 805 g) was obtained as a white solid in 68% yield.

^{1}H NMR (500 MHz, Water-d2) δ: 8.89(d, 2H), 8.77 (d, 2H) ppm.

Synthesis of *COOH-DS*

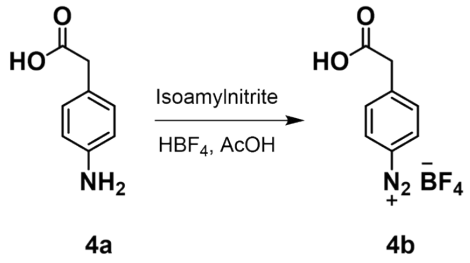

Scheme 4. Synthetic route for *COOH-DS* (4b).

4-(carboxymethyl)benzenediazonium tetrafluoroborate (4b). 2-(4-aminophenyl)acetic acid (5 mmol, 755.8 mg) and 48% v/v HBF_4 (2 mL) were dissolved in acetic acid (40 mL), isoamylnitrite (2 mL) was dissolved in acetic acid (20 mL) and was added dropwise. After 20 minutes Et_2O (20 mL) were slowly added until some turbidity appears. The mixture was cooled to -20ºC for 1h30' and the resulting precipitate was collected by filtration and washed with Et_2O. The compound (3.7 mmol, 937 g) was obtained as a white solid in 75% yield.

^{1}H NMR (500 MHz, Water-d2) δ: 8.89(d, 2H), 8.77 (d, 2H) ppm.

GFET fabrication

The fabrication process was employed for the fabrication of macro-GFET, 14 channels single-addressed GFET and 48-channels micro-GFET. A conventional lift-off process using the image reversal photoresist AZ5214E (Clariant, Germany) is followed in four-inch silicon wafers covered by 2 µm thick thermal oxide. The bottom metal layer is an e-beam evaporated Ti/Au, 10/100 nm in thickness. After graphene transfer, the graphene GFET active areas were defined by means of an oxygen-based reactive ion etching using a patterned HiPR 6512 resist mask which is subsequently removed in acetone. The patterning process of the top metal layer is similar to the bottom metal contact patterning, but the metal stack consisted in a Ni/Au, 20/200 nm thick film. Finally, SU-8 negative photoresist (SU-8 2005, MicroChem, USA) is used to passivate the metal leads while defining the graphene channel and metal contacts openings.

Figure S1. AFM scratching SiO_2/CVDg

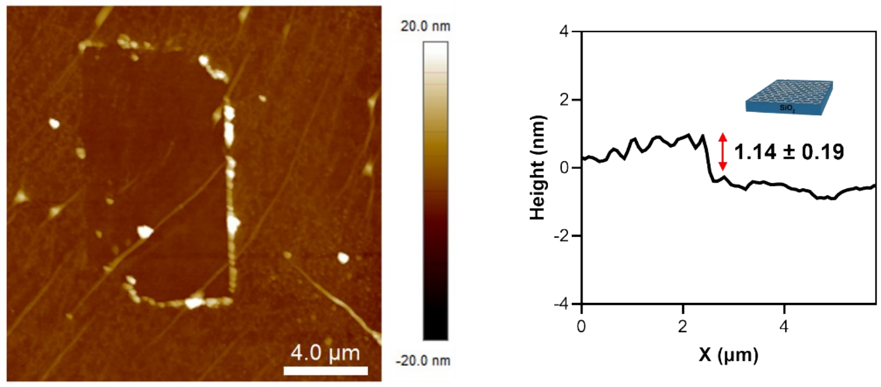

AFM scratching experiment performed on pristine SiO_2/CVDg.

Figure S2. AFM micrographs of SiO_2/CVDg functionalized samples

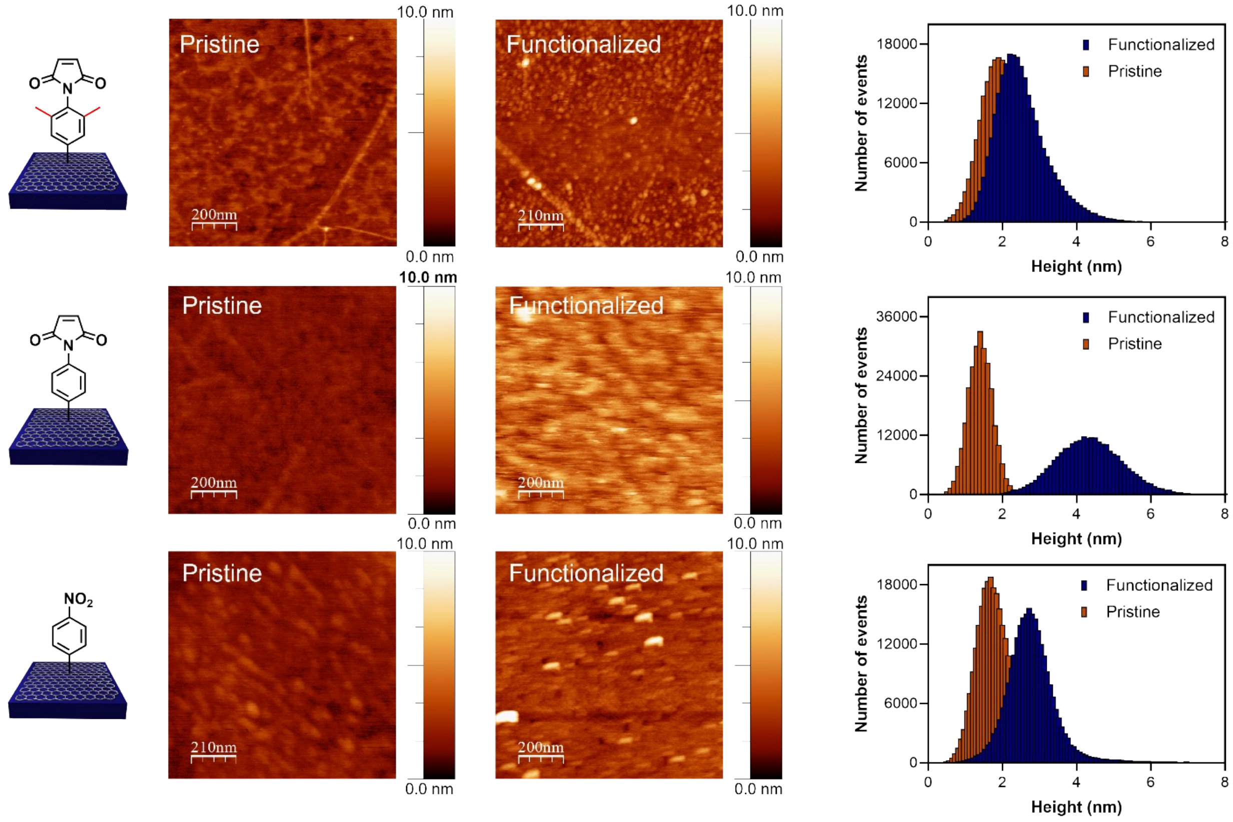

AFM images recorded before and after the functionalisation of SiO_2/CVDg-*mMAL*, SiO_2/CVDg-*MAL* and SiO_2/CVDg-NO_2. In the last column the roughness analysis performed on the images is included.

Figure S3. Raman spectra of SiO_2/CVDg functionalized samples

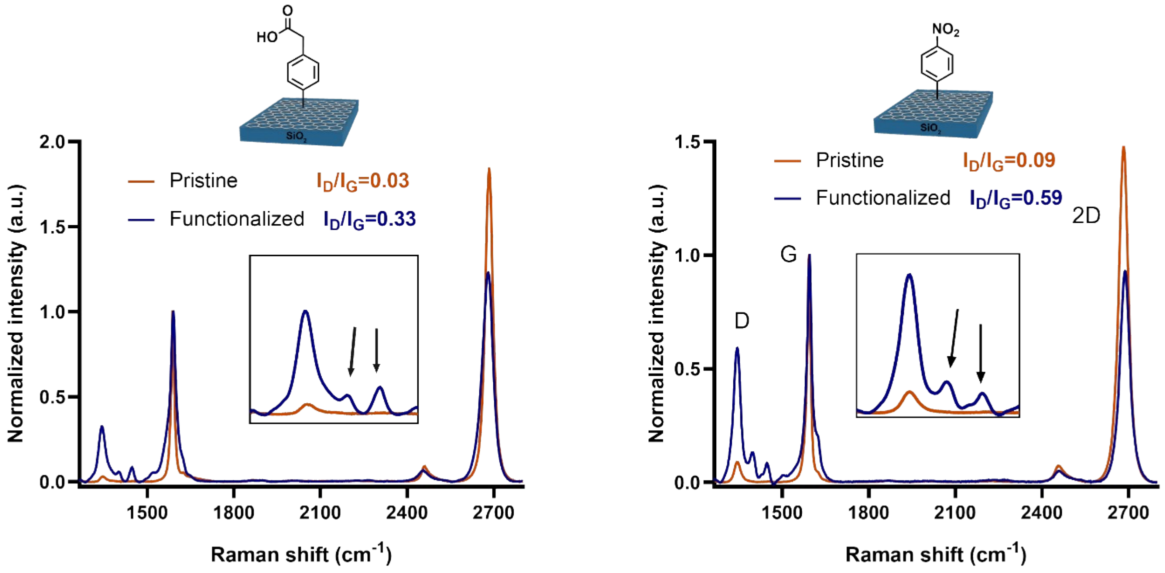

Raman spectra of SiO_2/CVDg before (orange) and after (blue) the functionalisation with *COOH-DS* (left) and *NO_2-DS* (right).

Figure S4. XPS characterisation of SiO_2/CVDg-*mMAL* and SiO_2/CVDg-*MAL*.

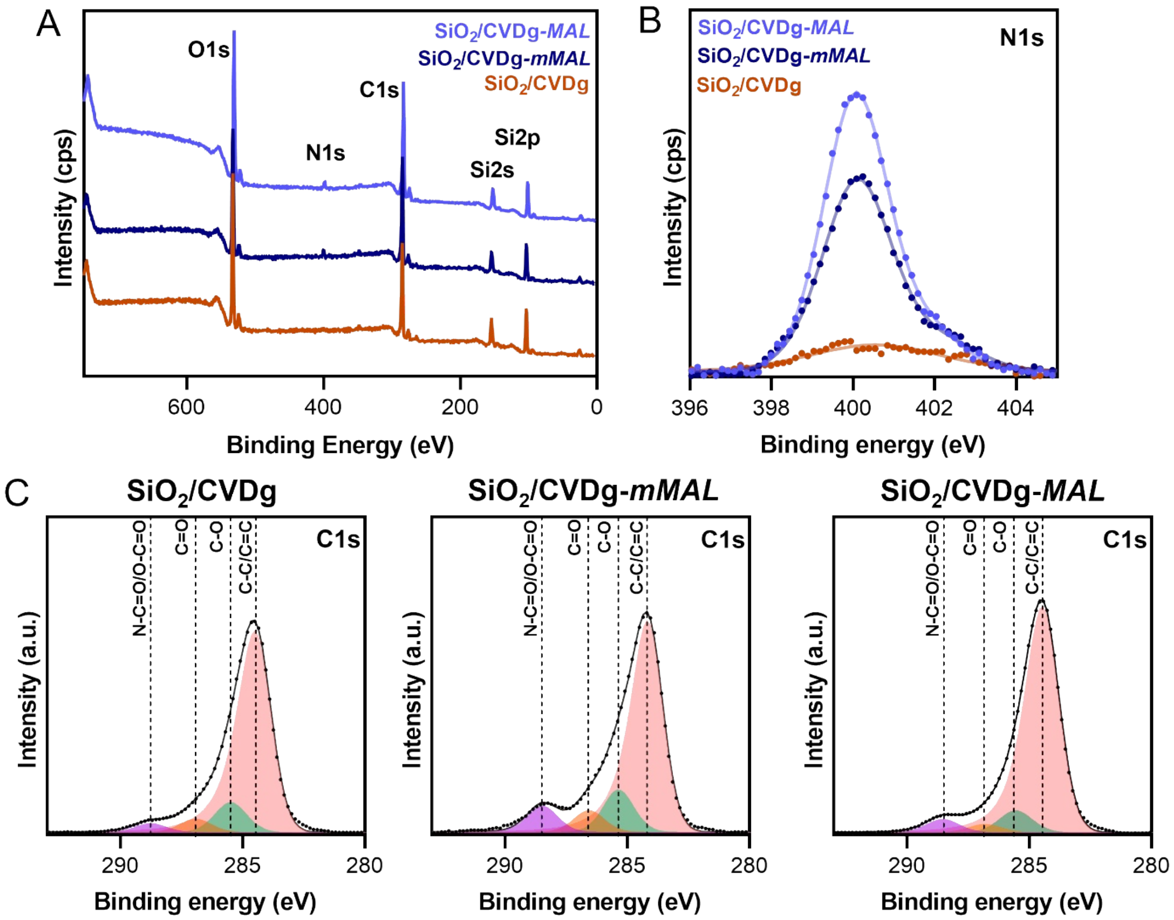

Survey spectra (A), high resolution N1s (B) and C1s (C) of pristine (orange), SiO_2/CVDg-*mMAL* (dark blue) and SiO_2/CVDg-*MAL* (light blue).

Table S1. XPS analysis of SiO_2/CVDg-*mMAL* and SiO_2/CVDg-*MAL*

Sample ID	%at survey regions			%at C1s components			
	N1s	C1s	O1s	C-C/C=C	C-O	C=O	N-C=O
SiO_2/CVDg	0.2	28.2	71.6	78.9	12.0	3.6	5.4
SiO_2/CVDg-mMAL	1.4	62.1	36.4	69.8	14.2	8.7	7.3
SiO_2/CVDg-MAL	2.0	65.8	32.2	83.5	8.3	3.2	5.0

XPS calculated atomic percentage of survey regions and C1s components for SiO_2/CVDg pristine, *SiO_2/CVDg-mMAL* and *SiO_2/CVDg-MAL.*

Figure S5. Characterisation of macro-GFET functionalized by CV.

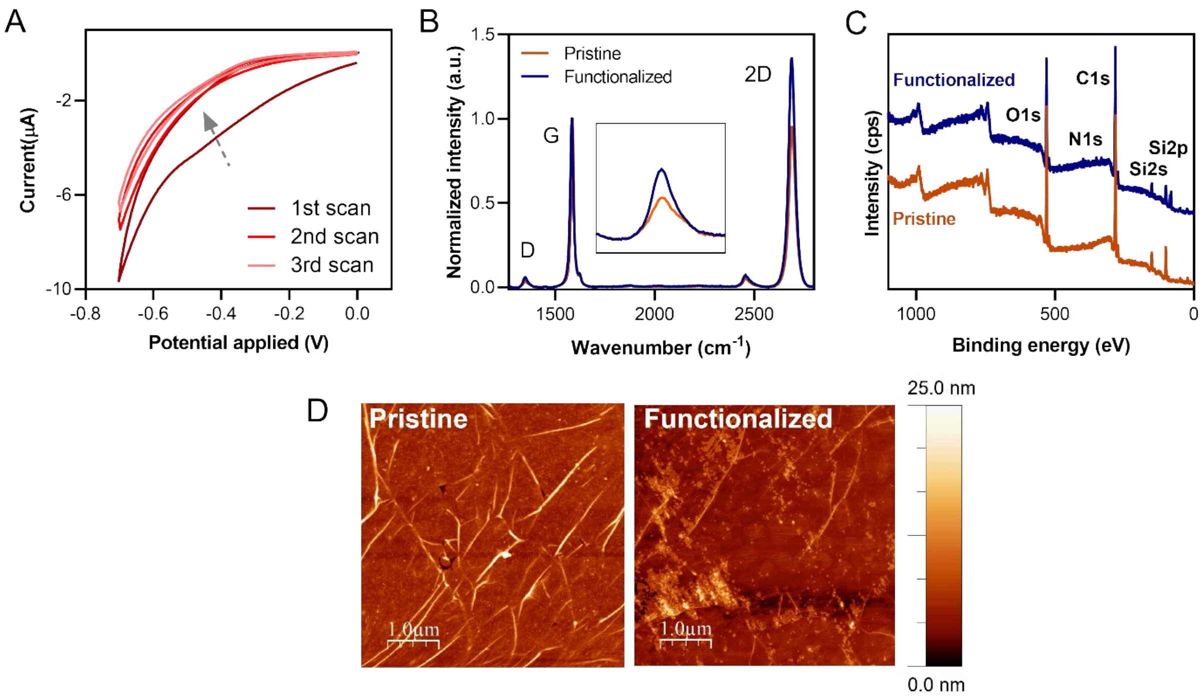

Characterisation of macro-GFET functionalized by cyclic voltammetry. A) Cyclic voltammetry performed in 5mM *mMAL-DS* solution in H_2O/EtOH (0.1 M KNO_3), scan rate 0.05 V s^{-1}. The dashed grey arrow shows the direction of reduction peak evolution over subsequent cycles. It should be noted that the low definition of the reduction peak of the diazonium salt is due to the presence of an insulating material such as SiO_2 as a support substrate for graphene. B) Raman spectra recorded before and after the functionalisation. C) XPS analysis performed before and after the reaction. D) AFM images recorded before and after the reaction.

Figure S6. Raman characterisation of mMAL-macro-GFET

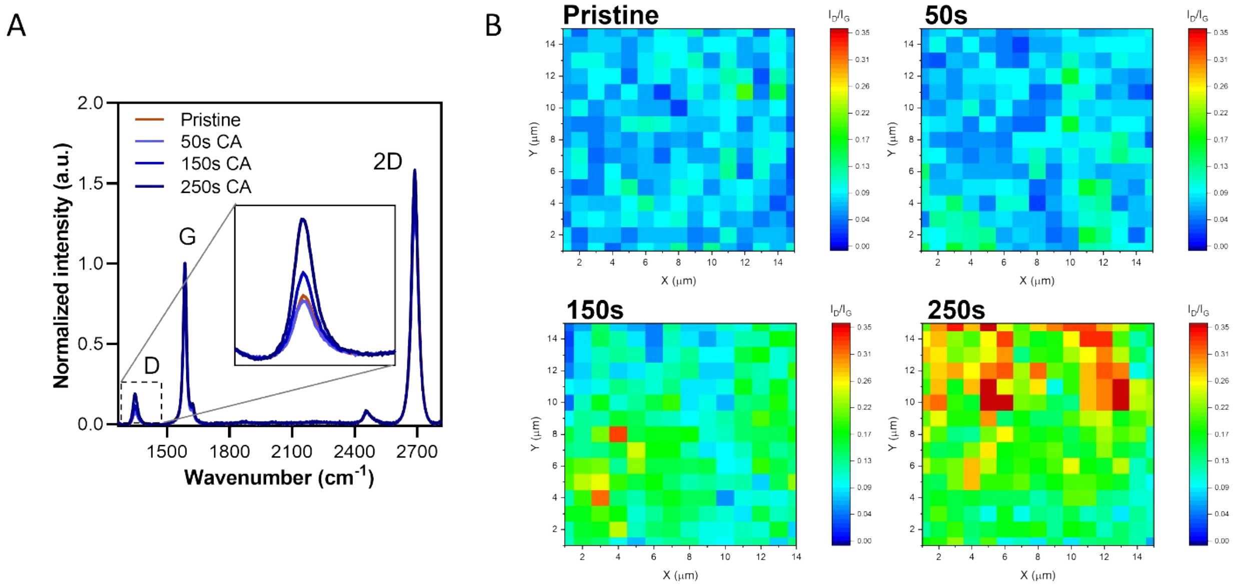

Raman spectra (A, average) and (B) mapping performed of macro-GFET on pristine material and after 50s, 150s and 250s of chronoamperometry in presence of mMAL-DS solution.

Figure S7. Transfer curve of macro-GFET.

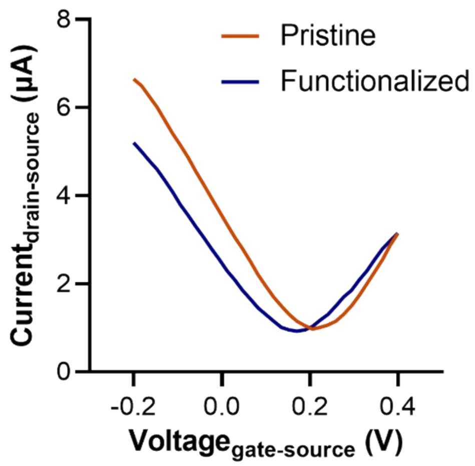

Transfer curve recorded before and after the functionalisation of macro-GFET with mMAL-DS (PBS 10mM, gate: Ag/AgCl (KCl 3M)).

Figure S8. XPS characterisation of *mMAL*-macro-GFET.

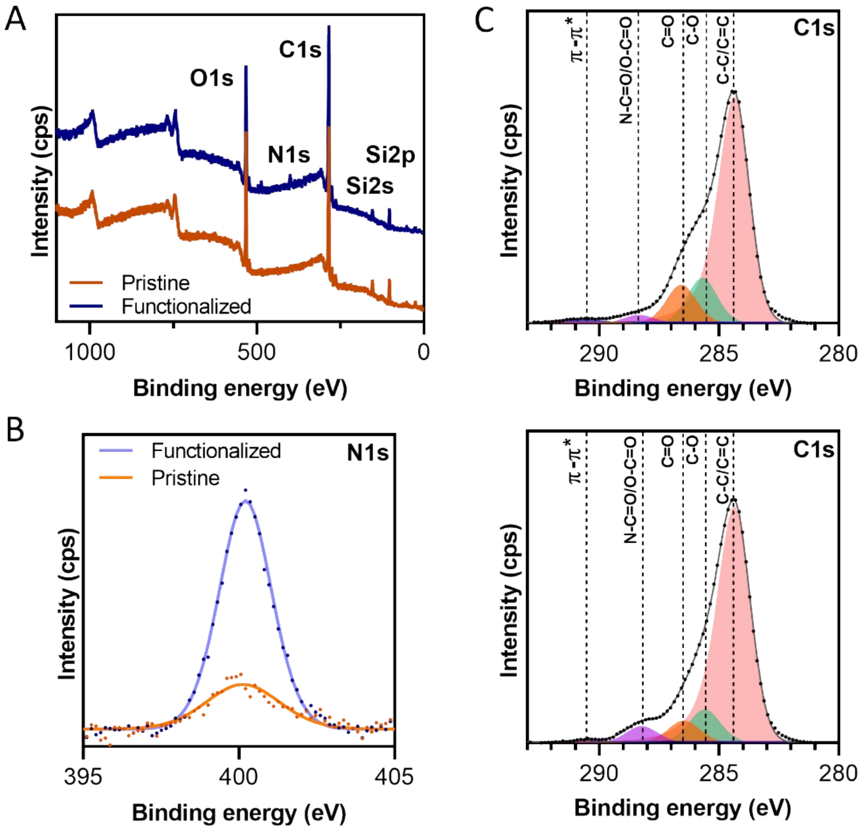

XPS analysis performed on macro-GFET before and after the functionalisation. A) Survey spectra, High resolution spectra of N1s (B) region and C1s (C) before (up) and after (down) the reaction.

Table S2. XPS calculated at% of *mMAL*-macro-GFET.

	at% pristine	at% funct
N1s	0.7	2.3
C1s	76.1	77.9
O1s	23.2	19.8

XPS calculated atomic percentage for macro-GFET pristine and functionalized.

Figure S9. Surface coverage quantification

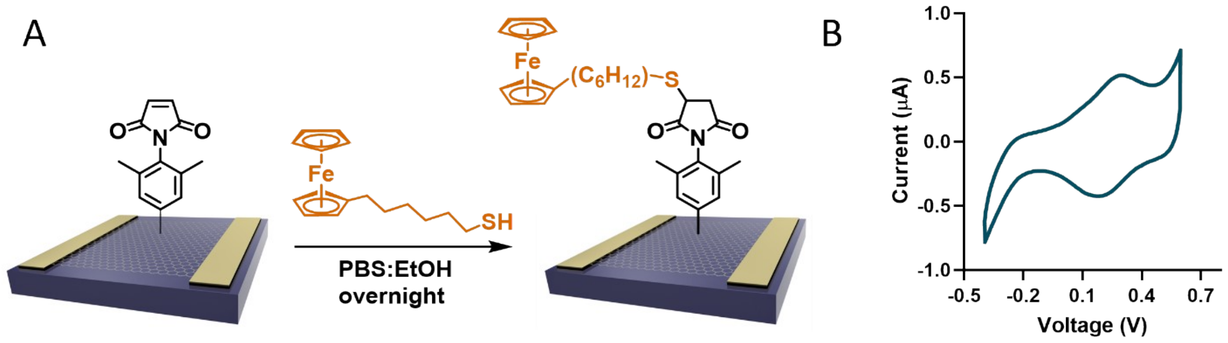

Schematic representation of Ferrocene-SH (Fc-SH) conjugation on functionalized macro-GFET (A) and resultant cyclic voltammetry used to calculate the surface coverage (B).

Surface coverage calculation

The surface coverage (Γ) was calculated using the Equation S1:

$$\Gamma = \frac{Q}{nFA} \# Eq. S1$$

where n is the number of exchanged electrons (1), F is the Faraday constant, A is the transistor surface area (0.36 cm^2) and Q is the anodic charge calculated by integrating the anodic peak of cyclic voltammetry performed on Fc-SH modified macro-GFET (Figure S9.B).

Figure S10. Charge carrier mobility of micro-GFET.

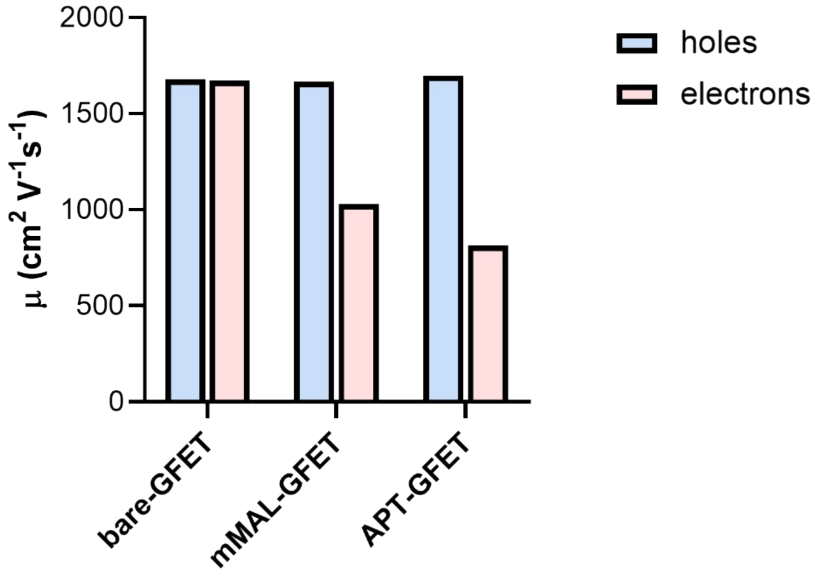

Mobility of graphene of micro-GFET after each functionalisation step divided in holes and electrons mobility.

Mobility calculation

The graphene mobility (μ) calculation was performed by means of the Equation S2:

$$\mu = slope \frac{L}{W \cdot V_{DS} \cdot C} \#Eq. S2$$

where L and W are the length and width of the channels (0.05 and 0.05 cm), V_{DS} is the fixed voltage between drain and source (0.05 V), C is the graphene capacitance (2 10^{6} F cm^{-2}) and the slope is calculated by linear fitting of the left or right part of the transfer curve.

Figure S11. Flow cell used for the micro-GFET calibration experiments.

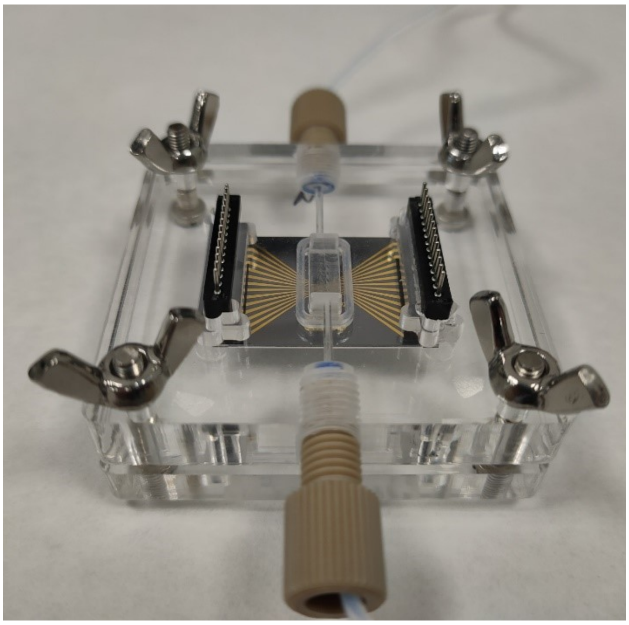

Figure S12. Calibrated response to serotonin addition on GFETs array.

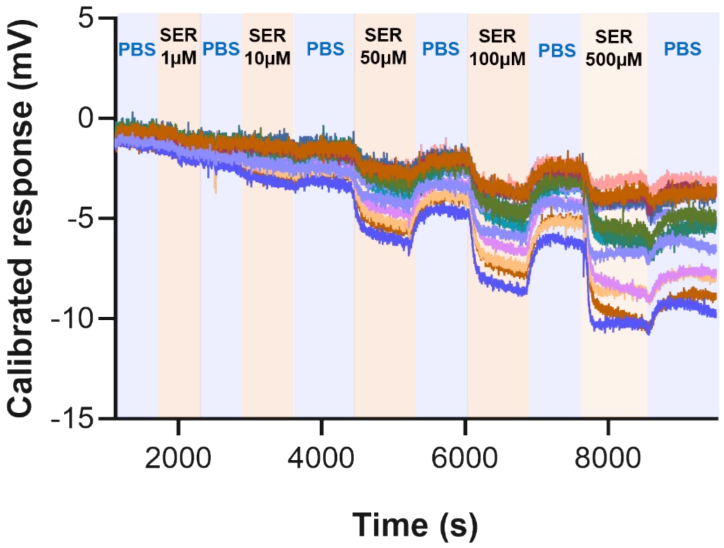

Calibrated voltage response of different channels under addition of different serotonin concentrations alternated with 10 mM PBS injections.

Debye length calculation

The Debye length can be expressed as follow:

$$\lambda_D = \sqrt{\frac{\varepsilon_0 \varepsilon_r k_B T}{2 N_A e^2 I}} \# Eq. S3$$

λ_D is the Debye length ε_0 is the permittivity of free space ε_r is the dielectric constant k_B is Boltzmann's constant, T is temperature (Kelvin) Na is Avogadro's number e is elementary charge *I* is the electrolyte ionic strength.

Table S5. Calculation of I and λ_D for different buffer including PBS from Sigma Aldrich and artificial cerebrospinal fluid (aCSF) from ref [7].

Buffer	Ionic strength I (mM)	Debye length λ_D (nm)
1x PBS (10 mM)	164	0.74
aCSF	158	0.76

Limit of detection

The sensitivity (*S*) of the sensor, which is defined as $\Delta I_{DS}/ \Delta V_{GS}$, was calculated as the slope of the linear regression of the linear portion of the calibration curve (1-100 μM). This value was employed,

along with the noise (σ, standard deviation of the blank), to calculate the limit of detection (LOD) following Equation S4

$$LOD = \frac{3\sigma}{S} \# Eq. S4$$
